# Starch-Silane Structure and Its Influence on the Hydrophobic Properties of Paper

**DOI:** 10.3390/molecules27103136

**Published:** 2022-05-13

**Authors:** Tomasz Nowak, Bartłomiej Mazela, Konrad Olejnik, Barbara Peplińska, Waldemar Perdoch

**Affiliations:** 1 Faculty of Forestry and Wood Technology, Poznań University of Life Sciences, Wojska Polskiego 28, 60-637 Poznan, Poland; tomasz.nowak@poskladani.pl (T.N.); bartlomiej.mazela@up.poznan.pl (B.M.); 2 POSkładani.pl A.T. Nowak spółka jawna, ul. Władysława Nehringa 8 lok. 1, 60-247 Poznan, Poland; 3 Centre of Papermaking and Printing, Lodz University of Technology, Wolczanska 221, 93-005 Lodz, Poland; konrad.olejnik@p.lodz.pl; 4 NanoBioMedical Centre, Adam Mickiewicz University, Umultowska 85, 61-614 Poznan, Poland; barp@amu.edu.pl

**Keywords:** silylated starch, hydrophobic agent, starch hydrophobization, paper coating, cellulose

## Abstract

Starch is an inexpensive, easily accessible, and widespread natural polymer. Due to its properties and availability, this polysaccharide is an attractive precursor for sustainable products. Considering its exploitation in adhesives and coatings, the major drawback of starch is its high affinity towards water. This study aims to explain the influence of the silane-starch coating on the hydrophobic properties of paper. The analysis of the organosilicon modified starch properties showed an enhanced hydrophobic behavior, suggesting higher durability for the coatings. Molecules of silanes with short aliphatic carbon chains were easily embedded in the starch structure. Longer side chains of silanes were primarily localized on the surface of the starch structure. The best hydrophobic properties were obtained for the paper coated with the composition based on starch and methyltrimethoxysilane. This coating also improved the bursting resistance and compressive strength of the tested paper. A static contact angle higher than 115° was achieved. PDA analysis confirmed the examined material exhibited high barrier properties towards water. The results extend the knowledge of the interaction of silane compositions in the presence of starch.

## 1. Introduction

Starch, along with cellulose and lignin, is perceived as one of the most attractive biopolymers due to its properties, availability, and low price. Starch is a polysaccharide of plant origin. Sources include roots (e.g., sweet potatoes, tapioca), tubers (e.g., potatoes), stems (e.g., sago palms), cereal grains (e.g., corn, rice, wheat, barley, oat, sorghum), and legumes (e.g., peas and beans), among others [1]. Starch has thermoplastic properties, is biodegradable, is soluble in hot water, and does not create technological obstacles in the recycling process [2]. However, poor mechanical properties, high moisture sorption, and brittleness are disadvantages that limit its industrial use as a stand-alone material [3,4]. Nevertheless, starch, subjected to an appropriate chemical treatment, possesses film-forming properties [2,5] that are used, for example, to control the absorbency of the paper surface. As a result, it improves the printing properties [6].

However, available methods of starch usage in the industry do not guarantee sufficient hydrophobization of paper products used in high moisture conditions, especially transport packaging [7]. Spiridon et al. [8] modified starch microparticles (TA-SM) with tartaric acid. They used the dry preparation technique in which the TA-SM microparticles were introduced as fillers within glycerol plasticized-corn starch. The modified starch was used as a coating agent. The water resistance and thermal stability were slightly improved through the addition of a large amount of cellulose due to the inter-component H-bonding between components. The evaluation of the mechanical properties revealed a significant increase in the tensile strength of the composites with increasing cellulose content [6].

The use of the starch properties to create colloidal solutions in hot water and its film-forming properties to evenly distribute compounds with hydrophobic characteristics, such as organosilicon compounds on the surface of biodegradable materials, appears to be a sustainable method of hydrophobization [9,10,11]. The search for synergy and compensation of the flaws between biopolymers and organic compounds seems to be the right direction for developing new industrial technologies [12]. Organosilicon compounds are used with great success in the hydrophobization of wood [13,14]. In hydrolysis, alcoholysis, condensation reactions, radical reactions, and sol-gel type reactions, bonds between hydroxyl groups in cellulose and organic substituents of alkoxysilanes are created [15,16,17,18]. These bonds are permanent and make the material significantly resistant to water activity. In terms of the chemical structure, starch is a polymer similar to cellulose. It is built out of repeating glucose units, combined with α-1,4-glycosidic (amylose) bonds and fractions of amylopectins that have branches, due to the presence of α-1,6-glycosidic bonds [19]. Its main difference from cellulose is the orientation of the glucose rings in the chain of amylose, and this seemingly minor difference is why starch, unlike cellulose, dissolves in hot water, creating a colloidal solution. The viscosity of the solution can be controlled by changing the concentration, which makes it easier to distribute it on a carrier in the form of a thin film [13,20].

Surface sizing and creating a film on a cellulose carrier is one of the most common operations used in the paper industry [19,21]. In terms of internal sizing to increase starch retention in paper, it is advisable to use cationic starch, which is characterized by a higher affinity to cellulose fibers [22]. Starch solutions can also be modified by adding various substances for papermaking purposes [19,21,22]. Furthermore, the binding and film-forming properties of starch create, for example, the possibility of applying alkoxysilanes on the surface of paper products. The hydroxyl groups of the glucose repeating units give the possibility of creating starch-silane bonds first, and subsequently, after applying on the paper substrate, starch–silane–cellulose bonds are created. That way, a consolidated hydrophobic layer is created.

There are known methods of paper hydrophobization using alkoxysilanes with substituents (e.g., isocyanate [23,24] or chlorine [25]), which guarantee the creation of silane-cellulose bonds. Several solutions in the literature present organosilicon compounds as a hydrophobizing agent for starch. The solution is known from the Glittenberg reports [22], in which a powdery starch was mixed with alkali silanes in the presence of alkali metal salts. In its description, granular starch was first treated with an aqueous solution of alkali metal salts of alkali silicates, such as sodium methyl-silicate, and subsequently air-dried at room temperature. The effect of the starch modification was the increase in the hydrophobicity of the dried granules. In addition, the new granulated silicone starch had a neutral pH, and in the form of a dry powder, it had mobility and flow properties similar to fluids. Another beneficial feature of the product was that it was resistant to water activity at a temperature up to 50 °C. Adding modified starch to water at the temperature of its gelling (or close to it) resulted in the ability to create smooth pastes and dispersions. According to the invention, modified starch had increased durability properties.

The invention described by Satterly [26] presented the method of obtaining starch in a powdery form that has increased hydrophobic properties. Hydrophobization was reached as a result of adding water-soluble silicones. The hydrophobic mixture was prepared at a temperature lower than the temperature of starch gelatinization (<60 °C). Amort et al. [27] described the modification of starch by using silanes, which was conducted in the presence of alkali metal hydroxides or alkali metal aluminates to silane hydrolyzates. According to the authors, the modified starch was characterized by better processing properties than a corresponding unmodified starch. Chen et al. [28] examined starch-silane (γ-methacryloxypropyl trimethoxysilane) systems to describe their adhesive properties. The glue samples prepared using trimethoxies γ-methacryloxypropyl silane as a cross-linking agent had increased stability during storage and increased shear resistance of the weld. Furthermore, the addition of silanes improved the plastic properties of the weld. Wei et al. [29] described a nanocrystalline starch that was subjected to modification through organosilicon compounds to increase the hydrophobic properties of the final product. The measured contact angle of the modified nanocrystalline starch was three times higher than the contact angle measured for the unmodified starch. Two-stage modification of starch using vinyltrimethoxysilane and a copolymer of methyl methacrylate (MMA), butyl acrylate (BA), and 2,2,2-trifluoroethyl methacrylate (3FMA) significantly changed the properties of the material. The latex foils obtained showed an increased hydrophobicity, lower free surface energy, and higher thermal stability than the unmodified starch [30].

Jariyasakoolroj and Chirachanchai [31] modified starch with silanes and polylactic acid. Their study demonstrated the creation of permanent bonds between components of the reaction mixture, and the created products had an increased level of crystallinity, lower glass transition temperature, and a slight increase in the tensile strength. In the study by Sandrine et al. [32], they described the influence of chemical modification on the properties of hemp-starch composites subjected to an alkaline treatment using silanes. Those actions increased the mechanical properties of the produced composites, especially their rigidity. Ganicz et al. [33,34] showed a method of paper hydrophilization through water emulsion of triethoxymethylsilane as a paper coating. The authors examined the effects of the applied coatings on the paper’s tensile strength, tear index, roughness, air permeance, and brightness.

The methods described above demonstrate the great potential of starch as a natural polymer, providing excellent opportunities within the scope of its chemical modification, especially in the range of conferring hydrophobic properties. However, the mentioned solutions do not apply to phenomena and interactions occurring between silanes and starch. In the literature, there is still a lack of complex studies regarding the influence of modified starch on the properties of cellulose material. For this reason, our study aims to examine the physicochemical interactions using Scanning Electron Microscopy (SEM) with Energy Dispersive X-Ray (EDX) analysis and hydrophobic properties (contact angle, Penetration Dynamics Analysis—PDA) between starch and organosilicon compounds in their application on a paper as a cellulose matrix. This study aims to explain the influence of the structure of the silane-starch coating on the hydrophobic properties of paper.

## 2. Results and Discussion

### 2.1. Water Penetration Dynamics of Modified Starch Applied on a Paper

**Figure 1:**
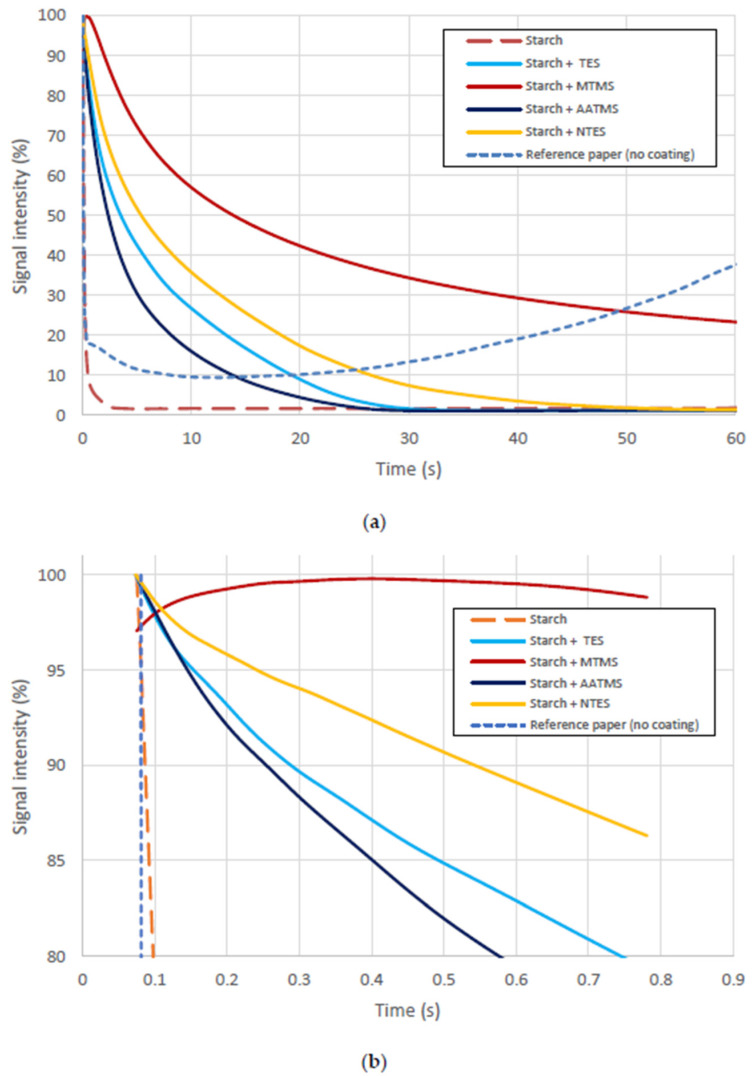
Analysis of PDA—the intensity of ultrasonic waves as a function of time for modified starch applied on a paper; (**a**) 0–60 s; and (**b**) 0–0.8 s.

The liquid–paper interaction is a very complex phenomenon. This process can be divided into two main stages: wetting and penetration. During the wetting stage, the water contact with the paper surface is hampered due to an air film adhering to the surface and air presence inside the surface pores. After the wetting stage, water finally begins to enter the paper structure and displaces air from the pores. The internal specific surface area of paper and its thickness increase, and micro-bubbles of air are observed within the adsorbed water. These air micro-bubbles are scattering centers for ultrasonic radiation; thus, a lower intensity signal is transmitted through the sample. The ultrasonic signal decreases faster as the liquid penetrates faster into the paper. The results of the measurements of the dynamics of water penetration into the structure of the paper samples with the coatings applied and the reference paper (without the coating) are presented in Table 1 and Figure 1a,b. The process of wetting and penetrating liquids is described by several parameters. Parameter “t95” correlates to the wetting rate of the surface and it corresponds to the size and structure of the pores at the paper surface. Parameter “Max” is the information about the surface resistance against the water. The wetting stage lasts until time “Max”. The water penetration begins after time “Max” [35,36]. This is the indicator of hydrophobicity and the sizing degree of the paper surface. Moreover, the slope of the curve beyond the Max value can be considered the penetration speed (ΔI/Δt) [35,36]. In the presented research, the calculations were performed for two times: 0.2 s and 3 s. This was because the reference paper and the starch coated paper had such a high water absorbency that after 3 s, the water absorption process was finished. Therefore, in order to show the dynamics of water absorption, it was necessary to also use a time shorter than 0.5 s. On the other hand, papers coated with a hydrophobization agent absorbed water much slower. In their case, performing a calculation for a time base of 3 s allowed for better presentation of the water absorption rate. The results in Table 1 show that the reference paper and the paper with only a starch based coating showed no wettability resistance. The wetting period described by the Max parameter was 0 s, and the values of ΔI/Δt (water absorption speed) during 0.2 s were very high. For the samples with coatings based on various starch-silane compositions, the lowest absorption rate in 0.2 s was recorded for the paper with the methyltrimethoxysilane (MTMS) coating. In this case, the value was −9.6, which means that the wetting barrier has not yet been overcome for this paper. This is confirmed by the value of the Max parameter, which was 0.4 s. The values of ΔI/Δt calculated for a time of 3 s confirmed that for the reference paper and paper with only a starch coating, the absorption process has already been completed. On the other hand, paper with a starch and MTMS coating was characterized by the lowest water absorption rate (i.e., the highest hydrophobicity).

The results presented in the table are confirmed by the curves presented in Figure 1a,b. The curve shape for the reference paper (uncoated) is typical for highly hydrophilic materials. The increase in the signal after 10 s of the measurement means that water penetrated the material structure quickly and evenly and the structure of the material began to swell (Figure 1a). Different materials suppress ultrasonic waves to varying degrees depending on their sorption properties. Water sorption occurs the fastest for starch applied on paper and the slowest for the samples coated with the addition of tetraethoxysilane tetraethoxysilane (TES), n-octyltriethoxysilane (NTES), and MTMS. This observation indicates that MTMS provided the best hydrophobization effect against water for paper samples with a cellulose surface. As was stated for the results from Table 1, the results from the PDA analysis indicate that in the initial phase of contact with water (i.e., between 0 up to 1 s), the examined variants of paper samples exhibited various wetting properties (Figure 1b). The rapid decrease in the signal for the uncoated paper in the initial wetting phase indicates the lack of a hydrophobic barrier on the surface and its rapid wetting (Figure 1b). The paper coated with starch modified through MTMS displayed a decreased wettability and permeability towards water, which was reflected in the increase in the intensity of the ultrasonic waves in the initial phase of the experiment (before the Max time) and the slower decrease after the time Max. The water uptake test (Cobb60 method) confirms the high effectiveness of the starch + MTMS composition. Compared to paper without any coating (Reference paper), the absorbency of starch + MTMS coated paper was more than seven times lower. The high absorbency of the other tested samples indicates that coating compositions containing AATMS, NTES or TES are not suitable as hydrophobic agents in papermaking applications.

### 2.2. Water Repellency Effect of Modified Starch Applied on a Paper

**Figure 2:**
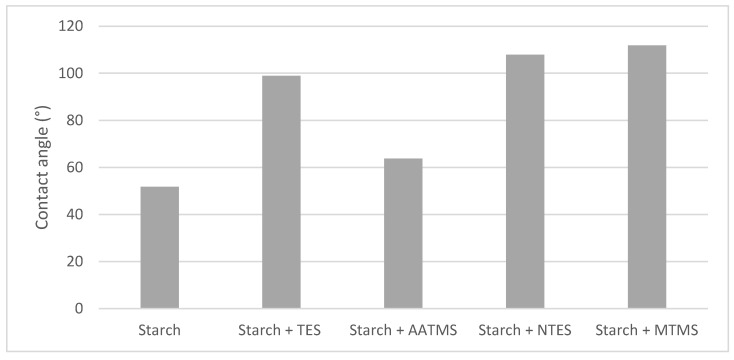
The static contact angle for modified starch applied on a paper.

The study of the water-barrier properties of the investigated paper samples was supported by the measurement of the contact angle (Figure 2). For the reference paper, the contact angle was not measurable due to the immediate penetration of water into the material structure. The four organosilicone modified starch (OMS) act as hydrophobizing agents but to a different extent, and in particular, MTMS and NTES enhance the contact angle over 100°, while *N*-(2-Aminoethyl)-3-aminopropyltrimethoxy silane (AATMS) is much less effective (63°). These results (Figure 2) suggest that the chemistry of MTMS and NTES comes from the hydrophobic alkyl chain. A chain that includes an amine structure, which is hydrophilic, does not protect a paper against water. The hydrophilic and hydrophobic nature of the alkyl part of silane probably influences the final results of the hydrophobic properties. Despite the low barrier properties demonstrated in the PDA study, starch applied on the paper slightly increased the static contact angle parameter (51°).

### 2.3. Effect of the Starch-MTMS Coating on the Selected Strength Properties of Paper Sheets

**Figure 3:**
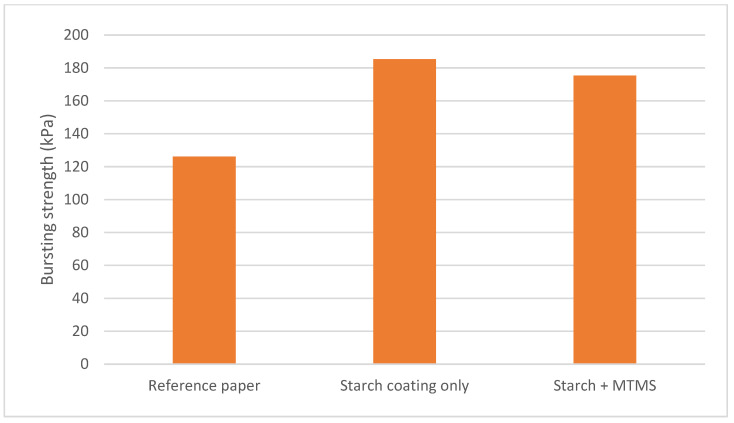
Comparison of the bursting strength for a reference paper, paper coated only with starch, and paper coated with a starch + MTMS composition.

Among all the starch and silane-based coating compositions tested, the best barrier effects were obtained for the coating containing MTMS. To investigate the effect of this coating on the mechanical properties of paper, proper tests were carried out. Typical strength properties that are typically determined for paper packaging materials were selected for this purpose: bursting strength and compressive strength. Figure 3 shows the comparison of the bursting resistance of a reference paper (uncoated), a paper with only a starch-based coating, and a paper with a coating containing starch and MTMS. The obtained results indicate that the application of the starch on the surface of the paper increased its bursting resistance. The coating based on the starch and MTMS mixture resulted in a reduction in the value of the bursting pressure by about 10 kPa in relation to the paper with the coating based on only starch. Despite the presence of MTMS, the bursting resistance was higher by approx. 60 kPa compared to the reference paper (without any coating). This means that the coating consisting of starch and MTMS still exhibits the properties that improve the bursting strength of paper.

**Figure 4:**
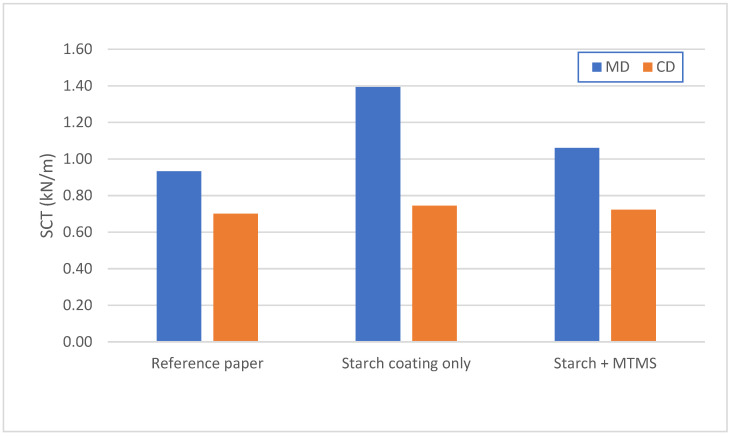
Comparison of the compression strength for a reference paper, paper coated with only starch, and paper coated with a starch + MTMS composition.

The results presented in Figure 4 show the compressive strength of the tested paper samples. In this case, the measurements were conducted for both directions of the paper (MD—Machine Direction and CD—Cross Direction). The obtained results confirm that the coating based on starch and MTMS does not adversely affect the strength properties of the tested paper.

### 2.4. Morphology Modification of OMS

**Figure 5:**
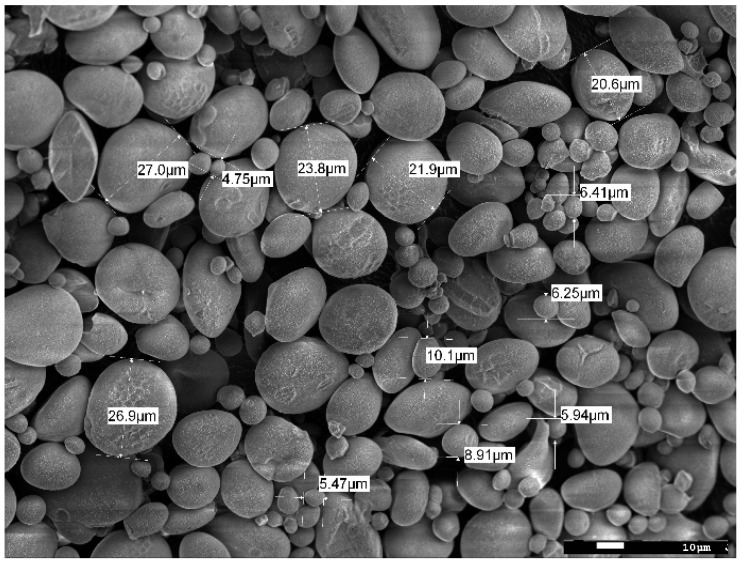
SEM micrograph of native starch granules ×600.

**Figure 6:**
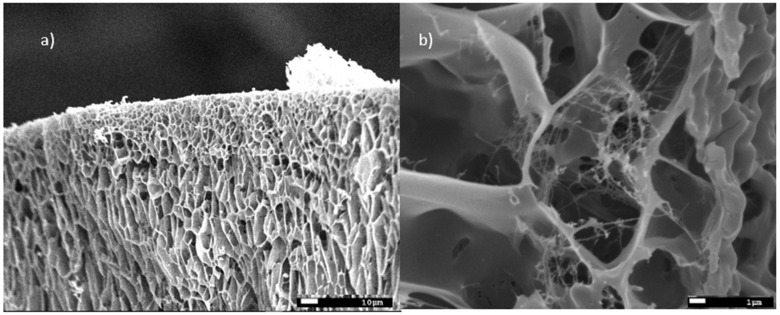
Cryo-SEM micrographs of an aqueous solution of starch (**a**) sample structure ×500; and (**b**) sample structure ×5000.

**Figure 7:**
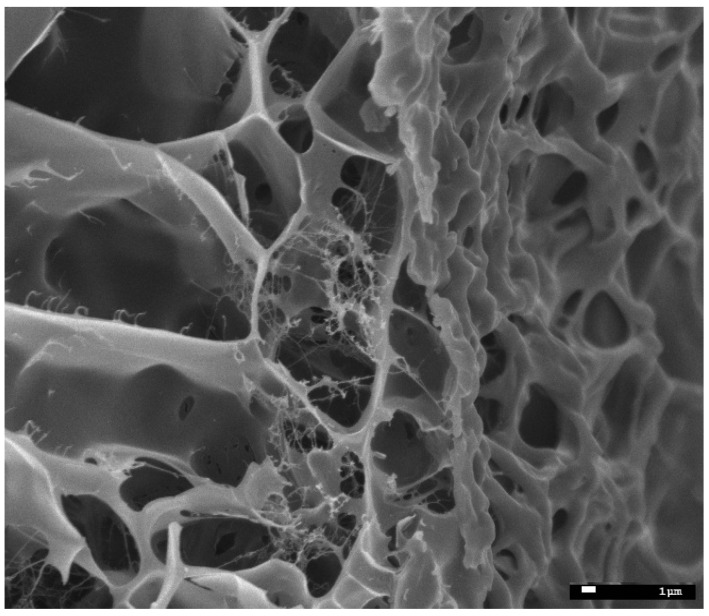
Cryo-SEM micrographs of an aqueous solution of starch ×3000—differences of the inside and surface structures.

The size of the starch granules subjected to gelatinization and modification varied and ranged between 4–30 µm (Figure 5). Starch subjected to thermal treatment in an aqueous solution (gelatinization process) and subsequently frozen out has a strongly porous structure (Figure 6a,b). It is worth noting that the examined material subjected to the physical treatment mentioned above presents a very different appearance of the surface structure and inside structure (i.e., cross-sectional area) of the sample. Those differences (Figure 7) are most likely the result of changes taking place in the starch gruel structure.

**Figure 8:**
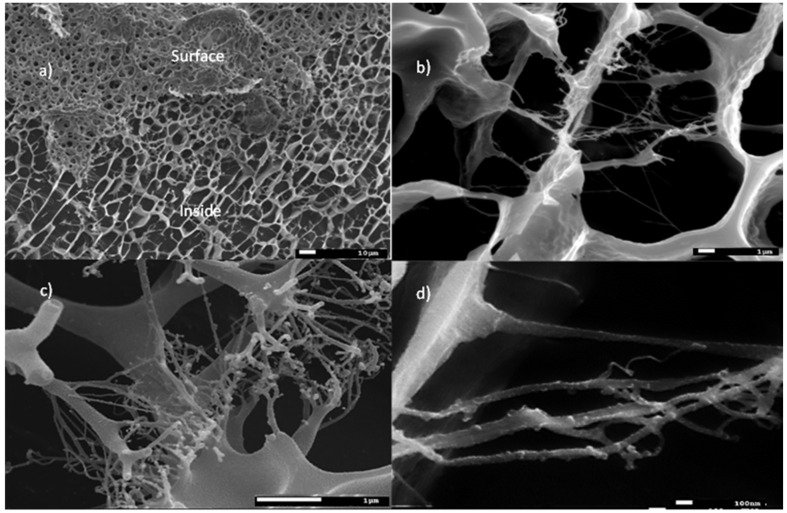
Cryo-SEM micrographs of an aqueous solution of starch modified through MTMS: (**a**) differences of the inside and surface structures ×500; (**b**) internal bonding in a modified starch structure ×5000; (**c**) internal bonding including silane groups ×20,000; and (**d**) internal bonding including silane groups ×50,000.

**Figure 9:**
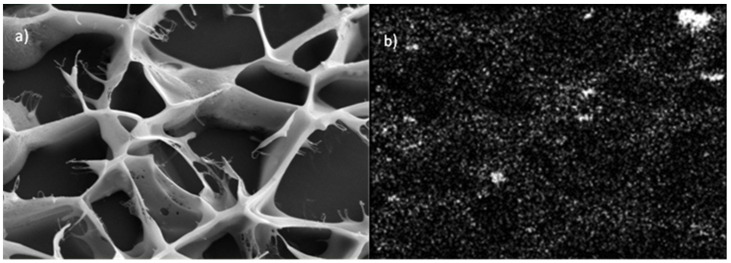
Cryo-SEM micrographs of an aqueous solution of starch modified through MTMS (**a**) and Si atom distribution (EDX mapping) in the starch structure (**b**).

Figure 8a–d summarizes the images of starch modified through MTMS. Similar to the case of unmodified starch gruel, a pronounced difference can be observed on the surface and inside the structure of the silane-modified material (Figure 8a). The microscopic image of starch modified by MTMS (Figure 8b) is similar to that of unmodified starch (Figure 6 and Figure 7). The examined material had a significantly higher stability than the structure of unmodified starch because the 10 kV energy used during the analysis allowed for the microscopic images with a magnification of 20,000 and 50,000 to be acquired without destroying the sample (Figure 8c,d). In comparison, the unmodified starch gruel degraded at 10 kV, i.e., during the analyses performed at a magnification of 5000. In Figure 6b, a cross-linking of the starch chains can be observed, at the end of which spherical structures are visible that contain a very high silicon content (according to EDX analysis), i.e., 2.35% by weight (1.27 atomic %). Organosilicon compounds were also embedded in the starch chain structure, which could be observed as distinct brighter spherical areas of the SEM micrographs of OMS through MTMS (Figure 8c,d). The silicon concentration in those areas was two times higher than in the remaining fragments of the cross-linked starch gruel. Following the EDX analysis, the mean content of MTMS in the gruel structure was 1.8% by weight (silicon is 1% of the weight in the structure of MTMS-modified starches). The most crucial information of the analysis of the structure of starch modified through MTMS was the confirmation that the added organosilicon compound had been distributed across all the cross-linked structures of starch. In Figure 9, the map of the silicon distribution in starch modified through MTMS is shown. The presence of silicon atoms precisely matches the structure of the starch gruel.

**Figure 10:**
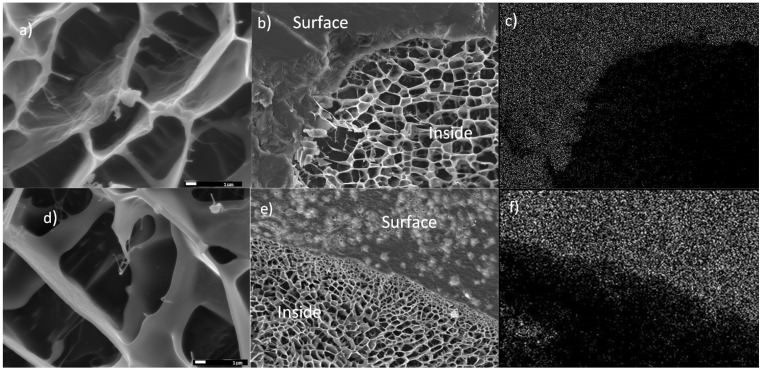
Cryo-SEM micrographs of starch modified through silanes: (**a**) internal bonding in an NTES modi-fied starch structure ×5000; (**b**) differences of the inside and surface structures with starch modified with NTES ×500; (**c**) EDX mapping of the (NTES) silicon distribution; (**d**) internal bonding in an AATMS modified starch structure ×5000; (**e**) differences of the inside and surface structures with starch modified with AATMS ×500; and (**f**) EDX mapping of the (AATMS) silicon distribution.

The modification of starch through NTES did not influence the change in the durability of the structure of the starch gruel. Similarly, in the case of unmodified starch, the structure of the examined material was degraded (Figure 10a) during the analysis (10 kV; magnification of 5000). In Figure 10b, an interesting structure was observed on the surface of the examined material. The EDX analysis (Figure 10c) confirmed that the sample surface is covered by silane. The silicon concentration on the surface of the examined material was 9.11% by weight (4.53 atomic %). There were no organosilicon compounds localized in the cross-section of the examined material. Therefore, NTES was not embedded in the starch structure, but only accumulated on its surface. Figure 10b shows the difference between the surface and cross-sectional structure of the examined material. The microscopic images allowed for the observation of characteristic, bright, spherical structures of silane embedded into the starch structure. EDX analysis allowed us to observe that AATMS in the starch structure accumulated mainly on the surface of the examined material. The mean concentration of silicon on the surface of the material was 5.03% by weight (2.69 atomic %), which was two times higher than the concentration of silicon measured in the inside layer of the sample (2.26% by weight; 1.23 atomic %). A significant fact is that more areas are visible with embedded organosilicon compounds in the surface layer than in the areas localized deeper, indicating limited penetration of the silane deep into the modifying polymer (Figure 10b–f).

The distribution of the organosilicon compound in the starch coating seems to be the limiting factor for the water-repellent properties of the paper. Therefore, the impact of the structure of the organosilicon compounds on their location in the starch–silane network should be considered first. From the chemical point of view, the properties of the components used in the research were diverse. MTMS, which hydrolyzes in the water environment, contributed to the uniform distribution of silicon in the structure of the aqueous solution of starch. It resulted in a highly hydrophobic surface formed on the cellulosic materials. The probable cause of the even distribution of silicon was also the small size of the silane molecule itself, the aliphatic chain of which contains only one carbon atom. The use of organosilicon compounds with longer aliphatic chains (NTES) or containing amino groups (AATMS), regardless of their solubility in water, formed a compact structure on the surface of the aqueous solution of starch. Notably, the production of a similar coating on the starch surface was not tantamount to giving the hydrophobic properties of cellulose` materials. The accumulation of starch-containing NTES sparingly soluble in water on the surface of the paper created a water barrier, which was confirmed by the results of the high contact angle and PDA analysis. The use of AATMS, which is soluble in water under the same conditions, did not increase the hydrophobic properties of the material.

## 3. Materials and Methods

### 3.1. Preparation of the Starch-Silane Hydrophobic Agent

Commercial wheat starch was used in the presented research (C*Flex 20002, Cargill, Incorporated, Minneapolis, MN, USA). The amylose content in the starch was 31.2 ± 0.16 wt%. The lipid content was 0.25 ± 0.02 wt% and the protein content was 0.30 ± 0.01 wt%. An aqueous solution of wheat starch (5% concentration) was stirred at 70 ± 5 °C in the presence of sodium hydroxide (0.25% *w*/*w*) to reduce the temperature of starch gelatinization. It also allowed the starch structure to loosen, as reported by other authors [37,38,39], to ease the penetration of silanes between the amylose and amylopectin chains. The suspension obtained was cooled to 40 ± 5 °C, and while mixing, organosilicon compounds were added (2.5% *w*/*w*). Organosilicons used in the current study are shown in Table 2.

Organosilicons used in the presented research have carbon chains of different lengths: methyltrimethoxysilane (MTMS), n-octyltriethoxysilane (NTES), *N*-(2-Aminoethyl)-3-aminopropyltrimethoxy silane (AATMS), and tetraethoxysilane (TES).

These four formulations were applied as a coating, using 100 g/m^2^ for cellulose paper (Whatman cellulose no. 3), by dipping it in the organosilicon solution (5 s) and letting it dry at room temperature for 48 h. Morphological and chemical characterization was performed to understand the hydrophobization mechanism.

### 3.2. Evaluation of Hydrophobic Properties

#### 3.2.1. Water Penetration Dynamics Analysis

Water penetration dynamics for paper samples coated with the starch-silane coated cellulose was measured using the PDA apparatus, Module S 05 (Emtec Electronic GmbH). The hand sheets were cut into three samples, each with a size of 3 cm × 7 cm. Measurements were performed separately for each sample in demineralized water at 20 °C, following the standard procedure described by the manufacturer [40]. The following parameters were used: ultrasound frequency of 2 MHz, measuring diameter of 35 mm, measurement duration of 60 s. The result was an arithmetic average value calculated from the series of three measurements.

#### 3.2.2. Contact Angle Analysis

A PGX+ Goniometer from Testing Machines Inc. (New Castle, DE, USA) was used for the contact angle measurements. The tests were carried out according to the TAPPI T 458 standard method. The water drop volume was 4 μL.

#### 3.2.3. Water Uptake

Water uptake test was conducted according to ISO 535:2014 (Cobb method, Cobb tester, Lorentzen & Wettre, Kista, Sweden). Time of the test was 60 s.

### 3.3. Mechanical Properties of the Paper Samples

Compressive strength (SCT test) was conducted according to ISO 9895:2008 standard (Instron 5564 machine, High Wycombe, UK). Bursting strength was conducted according to ISO 2758:2001 (Mullen Burst Machine, Lorentzen & Wettre, Kista, Sweden).

### 3.4. SEM-EDX Analysis of the Modified Starch Coating

The surface morphology of the samples was examined by the SEM method with cryogenic preparation system (Cryo-SEM), which enables direct investigation of the sample in the vitrified state. Cryo-scanning electron microscopy effectively observes wet samples without causing drying artifacts. It preserves the “natural” internal structure of the sample. This is particularly true for samples in the life sciences, where structures will retain their original conformation only in their fully hydrated state. Images were taken using the JEOL JSM-7001F TTLS (JEOL Ltd., Tokyo, Japan) scanning electron microscope equipped with the PP3000T cryo-SEM preparation system, which allows the cryo specimen to be prepared, processed, and transferred into the SEM chamber. The samples were cryo-fixed by plunging them into sub-cooled nitrogen (nitrogen slush, temperature of about −210 °C) and transferred by the vacuum cryo-transfer shuttle to the preparation chamber mounted onto the SEM. Inside the preparation chamber, at −185 °C, the specimen was fractured to expose a fresh surface. Then, it was sublimated and coated with a thin platinum layer. Finally, the sample was loaded under vacuum into the SEM chamber, where it remained frozen during imaging on the cold-stage, cooled by a nitrogen carrier (−190 °C). The images of all samples were taken by applying an accelerating voltage of 10 kV and using a secondary electron (SEI) detector. Elemental analysis and cryo-SEM elemental mapping were performed using the energy dispersive microanalysis (EDX) mode of an X-ray-equipped SEM that also applied a voltage of 10 kV.

## 4. Conclusions

The interaction of water and cellulose coated with starch modified with organosilicon compounds depends primarily on the physical properties and structure of the organosilicon compound itself. Based on the research, it can be concluded that the distribution of silicon in the gelatinized starch network depends primarily on the length of the organic chain in the organosilane molecule. MTMS molecules, which have very short side chains (methyl group), were easily embedded in the starch structure. Silanes containing longer side chains (e.g., AATMS, NTES) were localized mainly on the surface of the starch structure. The distribution of silanes resulting from their size does not translate unequivocally into making the coated materials hydrophobic. The physical properties of the organosilicon compounds primarily determine these properties. Poorly soluble substances such as NTES increase the hydrophobic properties, and water-soluble substances such as AATMS do not improve these properties. An even distribution of silane in starch and the developed permanent MTMS-starch bond indicate that this composition may be considered as a new type of hydrophobic agent for papermaking purposes. Static contact angle analysis and PDA analysis confirmed the high barrier properties of the examined material towards water. NTES containing an octa-carbon side chain in its structure produced a thin film on the starch surface, which also increased the barrier property of the coated paper. Despite producing a thin film on the starch surface, silane containing hydrophilic amine groups in its side chain did not provide barrier properties to the paper.

## Figures and Tables

**Table 1:** Results of the measurements of water penetration into the structure of the tested paper samples. The coefficient of variation for all cases ranged from 6.4 to 11.7%.

	Reference Paper (Uncoated)	Paper Coated with Starch	Paper Coated with Starch + AATMS	Paper Coated with Starch + NTES	Paper Coated with Starch + TES	Paper Coated with Starch + MTMS
MAX	0.0	0.0800	0.0780	0.0777	0.0777	0.4003
t95	0.0	0.0817	0.1837	0.2583	0.1537	1.3593
ΔI/Δt (0.2 s)	253.7	303.1	13.7	18.6	11.2	−9.6
ΔI/Δt (3 s)	1.6	3.9	36.3	9.8	30.4	5.6
Water uptake (Cobb60), g/m^2^	154.5	135.8	133.5	119.1	128.2	21.5

**Table 2:** Organosilicons applied to modify starch.

Acronym	Name	CAS Number	Chemical Structure
MTMS	methyltrimethoxysilane	1185-55-3	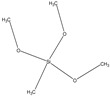
NTES	n-octyltriethoxysilane	2943-75-1	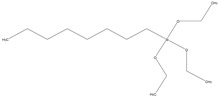
AATMS	*N*-(2-Aminoethyl)-3-aminopropyltrimethoxy silane	1760-24-3	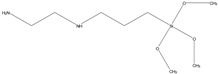
TES	Tetraethoxysilane (Tetraethyl orthosilicate)	78-10-4	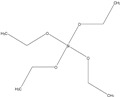

## Data Availability

The data presented in this study are available on request from the corresponding author. The data are not publicly available due to University policies.

## Sample Availability

Samples of the materials are available on request from the authors.

## References

[1] Swinkels J.J.M. (1985). Composition and Properties of Commercial Native Starches. Starch-Stärke.

[2] BeMiller J.N., Whistler R.L. (2009). Starch: Chemistry and Technology.

[3] Moriana R., Vilaplana F., Karlsson S., Ribes-Greus A. (2011). Improved Thermo-Mechanical Properties by the Addition of Natural Fibres in Starch-Based Sustainable Biocomposites. Compos. Part A Appl. Sci. Manuf..

[4] Ribba L., Garcia N.L., D’Accorso N., Goyanes S. (2017). Disadvantages of Starch-Based Materials, Feasible Alternatives in Order to Overcome These Limitations. Starch-Based Materials in Food Packaging.

[5] Jiménez A., Fabra M.J., Talens P., Chiralt A. (2012). Edible and Biodegradable Starch Films: A Review. Food Bioprocess Technol..

[6] Biricik Y., Sonmez S., Ozden O. (2011). Effects of Surface Sizing with Starch on Physical Strength Properties of Paper. Asian J. Chem..

[7] Larotonda F.D.S., Matsui K.N., Sobral P.J.A., Laurindo J.B. (2005). Hygroscopicity and Water Vapor Permeability of Kraft Paper Impregnated with Starch Acetate. J. Food Eng..

[8] Spiridon I., Teacă C.-A., Bodîrlău R., Bercea M. (2013). Behavior of Cellulose Reinforced Cross-Linked Starch Composite Films Made with Tartaric Acid Modified Starch Microparticles. J. Polym. Environ..

[9] Molavi H., Behfar S., Shariati M.A., Kaviani M., Atarod S. (2015). A Review on Biodegradable Starch Based Film. J. Microbiol. Biotechnol. Food Sci..

[10] Tai N.L., Adhikari R., Shanks R., Adhikari B. (2017). Flexible Starch-Polyurethane Films: Physiochemical Characteristics and Hydrophobicity. Carbohydr. Polym..

[11] Martinez-Pardo I., Shanks R.A., Adhikari B., Adhikari R. (2017). Thermoplastic Starch-Nanohybrid Films with Polyhedral Oligomeric Silsesquioxane. Carbohydr. Polym..

[12] Dal A.B., Hubbe M.A. (2021). Hydrophobic Copolymers Added with Starch at the Size Press of a Paper Machine: A Review of Findings and Likely Mechanisms. BioResources.

[13] Donath S., Militz H., Mai C. (2007). Weathering of Silane Treated Wood. Holz Roh-Und Werkst..

[14] Ratajczak I., Szentner K., Rissmann I., Mazela B., Hochmanska P. (2012). Treatment Formulation Based on Organosilanes and Plant Oil Blend—Reactivity to Wood and Cellulose. Wood Res..

[15] Xie Y., Hill C.A., Xiao Z., Militz H., Mai C. (2010). Silane Coupling Agents Used for Natural Fiber/Polymer Composites: A Review. Compos. Part A Appl. Sci. Manuf..

[16] Siuda J., Perdoch W., Mazela B., Zborowska M. (2019). Catalyzed Reaction of Cellulose and Lignin with Methyltrimethoxysilane—FT-IR, 13C NMR and 29Si NMR Studies. Materials.

[17] Tshabalala M.A., Gangstad J.E. (2003). Accelerated Weathering of Wood Surfaces Coated with Multifunctional Alkoxysilanes by Sol-Gel Deposition. J. Coat. Technol..

[18] Hill C.A., Farahani M.M., Hale M.D. (2004). The Use of Organo Alkoxysilane Coupling Agents for Wood Preservation. Holzforschung.

[19] Holik H. (2006). Handbook of Paper and Board.

[20] Jonhed A., Andersson C., Järnström L. (2008). Effects of Film Forming and Hydrophobic Properties of Starches on Surface Sized Packaging Paper. Packag. Technol. Sci. Int. J..

[21] Hubbe M.A. (2007). Paper’s Resistance to Wetting–A Review of Internal Sizing Chemicals and Their Effects. BioResources.

[22] Glittenberg D., Becker A. (1998). Cationic Starches for Surface Sizing. Pap. Technol..

[23] Cunha A.G., Freire C.S., Silvestre A.J., Neto C.P., Gandini A. (2010). Preparation and Characterization of Novel Highly Omniphobic Cellulose Fibers Organic–Inorganic Hybrid Materials. Carbohydr. Polym..

[24] Paquet O., Krouit M., Bras J., Thielemans W., Belgacem M.N. (2010). Surface Modification of Cellulose by PCL Grafts. Acta Mater..

[25] Cunha A.G., Freire C., Silvestre A., Neto C.P., Gandini A., Belgacem M.N., Chaussy D., Beneventi D. (2010). Preparation of Highly Hydrophobic and Lipophobic Cellulose Fibers by a Straightforward Gas–Solid Reaction. J. Colloid Interface Sci..

[26] Satterly K.P. (1963). Method of Rendering Starch Hydrophobic and Free Flowing.

[27] Amort J., Hanisch H., Klapdor U., van der Maas H., Suerken H.-P. (1985). Method for the Modification of Starch in an Aqueous Medium.

[28] Chen L., Wang Y., Fei P., Jin W., Xiong H., Wang Z. (2017). Enhancing the Performance of Starch-Based Wood Adhesive by Silane Coupling Agent (KH570). Int. J. Biol. Macromol..

[29] Wei B., Sun B., Zhang B., Long J., Chen L., Tian Y. (2016). Synthesis, Characterization and Hydrophobicity of Silylated Starch Nanocrystal. Carbohydr. Polym..

[30] Qu J., He L. (2013). Synthesis and Properties of Silane-Fluoroacrylate Grafted Starch. Carbohydr. Polym..

[31] Jariyasakoolroj P., Chirachanchai S. (2014). Silane Modified Starch for Compatible Reactive Blend with Poly (Lactic Acid). Carbohydr. Polym..

[32] Sandrine U.B., Isabelle V., Hoang M.T., Chadi M. (2015). Influence of Chemical Modification on Hemp–Starch Concrete. Constr. Build. Mater..

[33] Ganicz T., Olejnik K., Rózga-Wijas K., Kurjata J. (2020). New Method of Paper Hydrophobization Based on Starch-Cellulose-Siloxane Interactions. BioResources.

[34] Ganicz T., Rozga-Wijas K. (2021). Siloxane-Starch-Based Hydrophobic Coating for Multiple Recyclable Cellulosic Materials. Materials.

[35] Waldner C., Hirn U. (2020). Ultrasonic Liquid Penetration Measurement in Thin Sheets—Physical Mechanisms and Interpretation. Materials.

[36] Sarah K., Ulrich H. (2018). Short Timescale Wetting and Penetration on Porous Sheets Measured with Ultrasound, Direct Absorption and Contact Angle. RSC Adv..

[37] Kolpak F.J., Weih M., Blackwell J. (1978). Mercerization of Cellulose: 1. Determination of the Structure of Mercerized Cotton. Polymer.

[38] Okano T., Sarko A. (1985). Mercerization of Cellulose. II. Alkali–Cellulose Intermediates and a Possible Mercerization Mechanism. J. Appl. Polym. Sci..

[39] Tondi G., Wieland S., Wimmer T., Schnabel T., Petutschnigg A. (2012). Starch-Sugar Synergy in Wood Adhesion Science: Basic Studies and Particleboard Production. Eur. J. Wood Wood Prod..

[40] Grüner G. (1996). Emtec Penetration-Dynamics Analyzer.

